# The Central Mechanisms of Secretin in Regulating Multiple Behaviors

**DOI:** 10.3389/fendo.2014.00077

**Published:** 2014-05-21

**Authors:** Li Zhang, Billy K. C. Chow

**Affiliations:** ^1^School of Biological Sciences, University of Hong Kong, Hong Kong, China

**Keywords:** secretin, social behavioral, water and food intake, motor coordination and learning, neural transmission, neural development, knockout mice

## Abstract

Secretin (SCT) was firstly discovered as a gut peptide hormone in stimulating pancreatic secretion, while its novel neuropeptide role has drawn substantial research interests in recent years. SCT and its receptor (SCTR) are widely expressed in different brain regions, where they exert multiple cellular functions including neurotransmission, gene expression regulation, neurogenesis, and neural protection. As all these neural functions ultimately can affect behaviors, it is hypothesized that SCT controls multiple behavioral paradigms. Current findings support this hypothesis as SCT–SCTR axis participates in modulating social interaction, spatial learning, water and food intake, motor coordination, and motor learning behaviors. This mini-review focuses on various aspects of SCT and SCTR in hippocampus, hypothalamus, and cerebellum including distribution profiles, cellular functions, and behavioral phenotypes to elucidate the link between cellular mechanisms and behavioral control.

## Introduction

For the survival of animals, it is critical to control complex behaviors in a timely and precise manner via regulatory pathways including sensory inputs, integration in the central nervous system (CNS), and coordinated motor outputs to peripheral muscles. Within these modulatory processes, various neuroendocrine factors exert their roles. Classical neurohormones such as those from the hypothalamus–pituitary system (i.e., sex hormones, growth hormone, etc.) have been comprehensively studied regarding their behavioral effects. Moreover, a recently discovered group of “neuropeptides” began to show plausible neurophysiological functions. Among those, secretin (SCT) has been repeatedly reported by independent groups to modulate behavioral paradigms.

Secretin was initially considered to be a duodenum-derived chemical factor in stimulating pancreatic secretion (1). However, several studies in 1980s suggested its presence in the brain (2–4). The introduction of immunohistochemical (IHC) and *in situ* hybridization (ISH) staining discovered SCT and its receptor (SCTR) in multiple brain sites as summarized in Table 1. In adult brains, SCT and SCTR were: (1) expressed in hindlimb area of cerebral motor cortex and prominently distributed in hippocampus; (2) abundantly found in thalamus and hypothalamus; (3) not present in midbrains except for embryos; and (4) widely distributed in hindbrain regions including cerebellum and medulla oblongata. During embryonic development, transcripts and proteins of SCT and transcripts of SCTR were found in cerebellar primordium, tegmentum, and mesenchyme flexure as early as embryonic day 10.5 (5, 6). As these SCT- or SCTR-expressing neurons control unique behaviors (i.e., hippocampus: learning and memory; hypothalamus: sex, drinking, and feeding; cerebellum: motor coordination and motor learning), it was postulated that SCT had a role in multiple behaviors. The following part will describe past studies regarding the role of SCT in behavioral modulations.

**Table 1:** **Brain distributions of SCT and SCTR**.

Anatomical division	Subdivision	Neuron/neuron group	Expression profile	Reference
Telencephalon	Cerebral cortex	Pyramidal cell	SCT peptide^a^	(7)
	Hippocampus	DG, hilus, molecular layer	SCT gene^b^	(8)
		CA1	SCTR gene^b^	(9)
Diencephalon	Thalamus	Laterodorsal thalamic nucleus	SCTR gene	(10)
	Hypothalamus	SON, PVN, Arc	SCT + SCTR gene + peptide	(11–14)
	Posterior pituitary	Herring bodies in pars nervosa	SCT + SCTR peptide	(13)
	CVO	SFO, OVLT	SCT + SCTR gene + peptide	(15)
Mesencephalon	Midbrain		NSE^c^	(16)
Rhombencephalon	Cerebellum	Purkinje neuron, DCN	SCT gene + peptide	(17–20)
		Purkinje neuron, basket cell	SCTR gene	(17, 19)
	Medulla oblongata	NTS	SCTR gene	(10)

*^a^SCT peptide was found in pyramidal cells in cerebral cortex from humans, and colchicine-treated rats (7) but with less (11) or no forebrain expression (18) in untreated rats*.

*^b^SCT and SCTR gene expression was detected by staining the *lacZ* reporter*.

*^c^SCT and SCTR expression was found in embryonic mesencephalic neurons until birth (5, 6) but not in adults (16)*.

*All these cell-specific expression profiles have been reported previously using IHC (for peptide) or ISH staining (for gene expression) in intact rodents, unless otherwise specified*.

*DG, dentate gyrus; SON, supraoptic nucleus; PVN, paraventricular nucleus; Arc, arcuate nucleus; CVO, circumventricular organ; SFO, subfornical organ; OVLT, organum vasculosum of lamina terminalis; NSE, no significant expression; DCN, deep cerebellar nucleus; NTS, nucleus tractus solitarius*.

## SCT in Hippocampus Controls Social Behavior and Spatial Learning

Hippocampus has been well-known to be responsible for social behavior, memory, and spatial learning, all of which are dependent on neurogenesis and synaptic plasticity (21–23). Hippocampal neurons were found to express significant SCT and SCTR as described in Table 1. Functional evidences including activated adenylate cyclase (24) and increased secretion of neurotransmitter glutamate and gamma-aminobutyric acid (GABA) by SCT (25) further supported SCT in hippocampal regulated behaviors.

**Figure 1:**
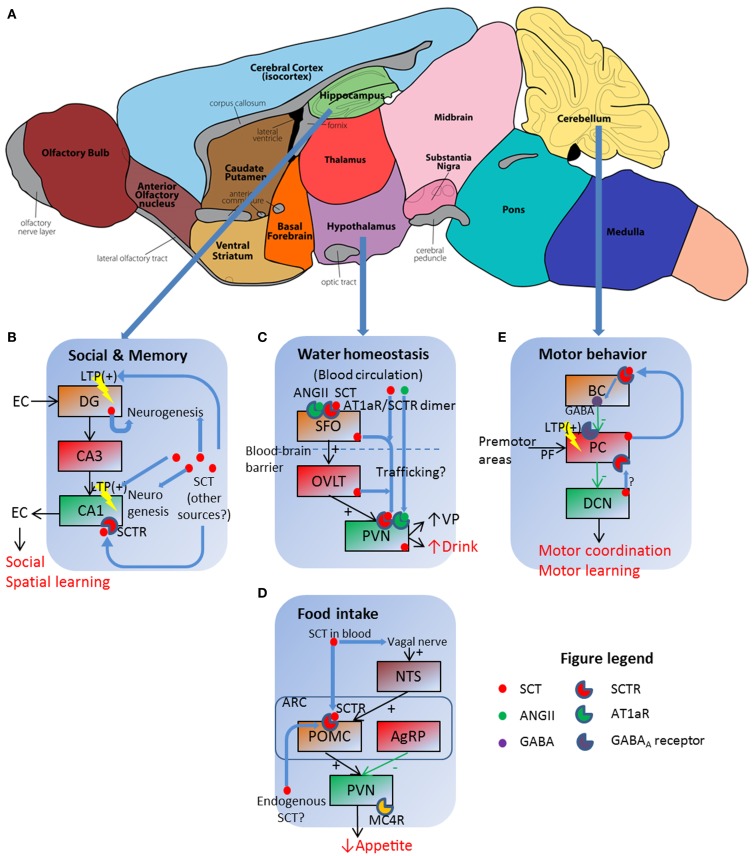
**Schematic illustration of SCT as a pleiotropic neuropeptide in regulating behavioral paradigms**. **(A)** Functional regions of a mouse brain, in which three sites with prominent SCT and SCTR expression are further illustrated in **(B–E)**. **(B)** SCT can modulate social and memory via both LTP control and neurogenesis in hippocampal DG and CA1 regions. EC, entorhinal cortex. **(C)** SCT works in conjunction with ANGII to exert a synergetic effect on hypothalamic SFO, which then relays the signal to PVN for the induction of water intake behavior. **(D)** Both vagal nerve and Arc neurons are responsive to SCT and further inhibit food intake via the activation of the melanocortin system. **(E)** SCT can induce presynaptic GABA release, and potentiate LTP in PF–PC synapse. BC, basket cell; PC, Purkinje cell; PF, parallel fiber. **(A)** is adapted from an illustration of the Gene Expression Nervous System Atlas (GENSAT) Project, NINDS Contracts N01NS02331 & HHSN271200723701C to The Rockefeller University (New York, NY) with permission.

One clinical trial reported that intravenous (IV) SCT injection improved eye contact, alertness, and expressive language ability on children with autistic spectrum disorders (ASD) (26). Replicated studies, however, had no significant effects (27) or only marginal improvements on some individuals (28–30), thereby rejecting SCT as an effective treatment against ASD (31). Although clinical studies did not get satisfactory results, animal experiments did reveal the role of SCT in social behaviors. A complete behavioral phenotyping in SCTR knockout (KO) mice reported impaired social interaction as shown by higher dominance percentage in a tube test and lower recognition ratio in a partition test (9). Such behavioral impairments were attributed to neuroanatomical and electrophysiological abnormalities: SCTR KO mice had fewer dendritic spines on CA1 pyramidal cells, in addition to impaired long-term potentiation (LTP) induction and maintenance (9). This synaptic plasticity dysfunction reoccurred in SCT KO mice which, however, had normal dendritic morphology (8). In behavioral studies, SCT KO mice had impaired spatial learning in the water maze task as they spent longer time to find the relocated hidden platform (32). In concurrent histological and electrophysiological examinations, higher apoptosis of neural progenitor cells in DG during postnatal development occurred, along with impaired LTP (32). In summary, SCT was involved in hippocampal neurogenesis and neural transmission including synaptic plasticity, all of which contributed to social behavioral and spatial learning as illustrated in Figure 1B.

## Hypothalamic SCT Stimulates Water Drinking

In the classical model for water homeostasis, vasopressin (VP) from posterior pituitary functions as an antidiuretic factor to induce renal water reabsorption while liver-originated angiotensin II (ANGII) has central effects stimulating VP release and water intake in hypothalamic neurons. Our research group for the first time reported polydipsia and polyuria in SCTR KO mice under both normal (33) and hyperosmolality conditions (34). Follow-up studies revealed that SCT was released from posterior pituitary following hyperosmolality stress to stimulate VP secretion (13). Therefore, the interruption of SCT–SCTR axis decreased VP secretion and led to lower renal water reabsorption, which further induced polyuria and stimulated water drinking as a consequence. We also measured central effects of SCT in drinking behaviors: intracerebroventricular (ICV) injection of SCT increased water intake in both wild type (WT) and SCT KO mice but not in SCTR KO ones (15). This potentiation, however, did not occur when SCT is peripherally injected (35). More importantly, ICV ANGII injection induced dipsogenic effects in WT mice but not in SCT KO or SCTR KO ones (15). This clearly suggested that ANGII exerted water intake control via SCT–SCTR-dependent pathways. Evidences supporting this model came from the co-localization of SCT and ANGII receptor type 1 (AT1aR) in hypothalamic PVN, along with the induction of *Sct* gene expression in SFO, OVLT, and PVN following centrally (15) but not peripherally injected ANGII (35). This sequential induction of *Sct* gene expression plus a higher Fos-immunoreactivity in SFO after ICV SCT injection (35) suggested that SCT firstly activated SFO, which then relayed the signal via OVLT to PVN to evoke drinking behaviors. In a word, SCT worked in conjunction with ANGII to stimulate water intake.

There were unsolved questions in the aforementioned model: ICV ANGII still induced water intake in SCT KO and SCTR KO mice, although at dramatically reduced levels compared to WT controls (15). This phenotype suggested that some SCT-independent pathways must exist although SCT-mediated ANGII regulation was more potent in terms of water intake control. This riddle has partially been elucidated in our recent study, which proposed a receptor heteromer between SCTR and AT1aR (34). As illustrated in Figure 1C, this receptor complex endowed strong synergistic effects to SCT and ANGII as low concentration of both peptides (10 ng ANGII, 50 ng SCT) had comparable dipsogenic phenotypes as those produced by 10-fold concentration of each peptide alone (34). In addition, we were able to totally abolish hyperosmolality-induced water intake via ICV injection of AT1aR transmembrane peptide-1 (ATM-1), which inhibited SCTR/AT1aR heteromer formation only but not SCTR/SCTR or AT1aR/AT1aR homomer on the cell surface (34). This working model indicated the potential *in vivo* role of SCTR/AT1aR heteromer in regulating water drinking behaviors as both receptors were co-expressed in the same hypothalamic neurons (15).

## SCT Suppresses Food Intake via the Melanocortin System

Previous studies reported appetite control by SCT but were somehow inconsistent. Peripheral SCT injection led to depressed food intake in both normal feeding (36) and fasted animals (37). Rat feeding pattern, however, was unaffected by intraperitoneally (IP)-injected SCT (38). Our research group recently reported a suppression of food intake in fasted mice by either IP or ICV SCT administration (14). This effect was determined to be SCTR-specific as SCTR KO mice did not exhibit such anorectic effects (14). Further studies revealed that both central and peripheral pathways existed under these feeding pattern changes. In hypothalamus, ICV injection of melanocortin-4 receptor (MC4R) antagonist SHU9119 reduced IP- or ICV-induced food intake suppression in WT and SCT KO mice (14). This information plus the SCT-stimulated expression of *Mc4r*, *Trh*, and *Pomc* gene (14), all of which are factors in melanocortin system for appetite control, clearly suggested that SCT works via the activation of melanocortin system to inhibit food intake (Figure 1D). SCT was believed to be endogenously released from hypothalamic neurons (12), although *in vivo* evidence was still lacked. In addition to this central mechanism, circulated SCT functioned via vagal afferent nerves as either surgical vagotomy or neurotoxin capsaicin treatment eliminated food intake suppression caused by IP- but not ICV-injected SCT (39). This is consistent with previous studies showing that peripheral SCT is able to activate vagal afferent and area postrema (AP) neurons (40). SCT induced Fos-immunoreactivity in NTS, AP, and dorsal vagal complex (DVC) after IP injection in intact mice but not in vagotomized or capsaicin-treated ones (39). Brainstem activation then stimulated POMC neurons in Arc, which is Fos-positive after IP SCT infusion (14). However, this effect was abolished when animals are vagotomized or capsaicin-treated (39). In summary, SCT either locally activated the melanocortin system in an auto-/paracrine manner or worked via vagal nerve to suppress food intake (see Figure 1D for a simplified working model).

Besides direct regulation on food intake, SCT may also participate in the regulatory network and cross-talk with other hormones to control the appetite. One possible candidate leptin, was synthesized from adipocytes and inhibited Arc neurons expressing neuropeptide Y (NPY) and agouti-related peptide (AgRP) whilst stimulated α-melanocyte-stimulating hormone (α-MSH), thereby suppressing food intake. The IV infusion of SCT increased plasma leptin levels (41), thereby inhibiting feeding behavior. It was further noticed that ICV-leptin elevated SCT expression in ventromedial hypothalamus (42). So SCT and leptin may work synergistically to exert the anorexic effect, as those for SCT and ANGII in water intake control. Such cross-talk may be further broadened, for example, cholecystokinin (CCK) can also synergistically interact with SCT at the vagal afferent nerve (43). More gastrointestinal hormones including glucagon-like peptide-1 (GLP-1), ghrelin, and amylin also activated certain SFO neurons (44). Thus SCT may collaborate with other gut peptides to form an integrated network modulating feeding behavior.

## SCT Regulates Motor Coordination and Motor Learning Behavior via the Facilitation of Purkinje Neuron Inhibitory Transmission

Motor effects of SCT were initiated by Charlton’s group, who discovered a lowered open-field activity and novel object approach after ICV injection of SCT (45), and a later one in which SCT was found to increase the latency of withdrawal jumping response (46). In SCTR KO mice, lower open-field activity and deficits of motor learning on rotarod were reported (9). Other groups showed that stereotypic circular movements in Japanese waltzing mice were attenuated by ICV or intranasal application of SCT, which improved horizontal movements but did not influence the explorative behavior (47, 48). One recent study suggested SCT in enhancing eye-blink conditioning, a classical cerebellar-related learning behavior (49). In summary, past researches provided knowledge about SCT’s neuropeptide function regarding motor behaviors. Nonetheless, systematic behavioral phenotyping was lacked, neither was the underlying mechanism.

Our research group for the first time developed a conditional SCT KO mouse model (Pur-SCT KO) in which *Sct* gene was specifically eliminated in cerebellar Purkinje neurons (20). We focused on Purkinje neurons because they regulated motor coordination and motor learning, as reported in Profilin 1 KO (50) and *tbl* mice (51). Motor behavioral genotyping in Pur-SCT KO mice showed significant impaired motor coordination and motor learning abilities (20): KO mice held a bar for a shorter time, spent longer climbing a wire mesh, and displayed insignificant improvements of rotarod latencies after repeated training. These abnormalities were replicated in SCT KO and SCTR KO mice (20), suggesting that Purkinje-derived SCT and SCT–SCTR axis were indispensable for motor behavioral controls.

This study supported the role of SCT in potentiating Purkinje neuron inhibitory transmission as previously reported (17). In this working model, SCT was endogenously produced from Purkinje neurons (52) following cytosolic calcium peak. It then functioned as a retrograde messenger, binding on presynaptic basket neurons, and induced inhibitory neurotransmitter GABA release (17). Other possible mechanisms still existed, however, as recent finding suggested that SCT suppressed intracellular trafficking of potassium channel Kv1.2 in both basket cell axonal terminals and Purkinje neuron dendrites (49). These reduced Kv1.2 ion currents led to presynaptic GABA release (53) and post-synaptic facilitation of parallel fiber–Purkinje neuron long-term depression (LTD) (54). Therefore, SCT worked via both pre- and post-synaptic pathways to modulate inhibitory transmission of Purkinje neurons (Figure 1E). In addition, SCT was also found in DCN (20), which were under inhibitory innervation of Purkinje neurons and sent output transmissions to premotor area in brain stem. Thus, SCT may mediate cerebellar transmission at multiple levels to accomplish motor behavioral control. Besides electrophysiological effects, SCT also affected cerebellar neurogenesis as that in hippocampal neurons. Supporting evidences included neural protection of cerebellar granular cell progenitors against ethanol toxicity (55) and behavioral phenotyping with later onset of cerebellar-related neural reflexes in Pur-SCT KO juveniles (20). Further studies are required to describe *in vivo* neural developmental profiles under the application or deprivation of SCT.

## Future Perspectives: The Full Paradigm of SCT in Neurobehavioral Regulations

Our current knowledge has established SCT as a pleiotropic neurohormone in behavioral modulations as summarized in Figure 1. These studies, however, are far from complete as SCT’s entry routes to CNS, its functioning sites, and cellular mechanisms largely remained unknown. As mentioned above, peripheral SCT can directly stimulate CVO neurons without crossing the blood–brain barrier (BBB) or activating vagal afferent to inhibit food intake. On the other hand, the possibility of SCT to cross the BBB has also been reported (56) by transmembrane diffusion (57). In addition to peripheral sources, SCT is produced also from multiple central neurons, for example, in hypothalamus (12) and cerebellum (52). Within the CNS, SCT may reach its target sites by axonal transport. The multiple routes of SCT in affecting central functions are supported by the observation that either of ICV, IV, IP, or even intranasal administration of SCT (48) could induce behavioral changes.

Secretin’s precise functioning sites and mechanisms can be investigated by more behavioral experiments, conditional KO animals, and *in vivo* neurophysiological studies. Firstly, the more robust and site-specific behavioral test can help to locate SCT-mediated neurons. One example is eye-blink conditioning, which is closely related to LTD at parallel fiber–Purkinje cell synapse (58). Secondly, *Sct* or *Sctr* gene can be turned-off in a spatial- or temporal-specific manner by various Cre–Loxp models. As each behavior involves multiple neurons, cell-specific KO models can better elucidate sources and functional sites of SCT. Temporal-specific KO models, on the other hand, play an irreplaceable role in studying developmental effects of SCT. Lastly, we recommend the usage of *in vivo* electrophysiological and imaging techniques to study the real-time neural activity when animals are performing behavioral tasks. This should help us to establish more valid link between cellular pathways and behavioral phenotypes. By all these advanced methods, behavioral paradigms modulated by SCT could further be elaborated. These results can help us to better understand neurobehavioral modulations and to develop potential drug candidates against various behavioral disorders.

## Conflict of Interest Statement

The authors declare that the research was conducted in the absence of any commercial or financial relationships that could be construed as a potential conflict of interest.
